# A Novel Student Engagement Analysis of Real Classroom Teaching Using Unified Body Orientation Estimation †

**DOI:** 10.3390/s25206421

**Published:** 2025-10-17

**Authors:** Yuqing Chen, Jiawen Li, Yixin Liu, Fei Jiang

**Affiliations:** 1 Shanghai Institute of Artificial Intelligence for Education, East China Normal University, Shanghai 200062, China; 52265901026@stu.ecnu.edu.cn (Y.C.); jwli@stu.ecnu.edu.cn (J.L.); 2 School of Computer Science and Technology, East China Normal University, Shanghai 200062, China; 51265901107@stu.ecnu.edu.cn; 3 Data Element Center, Chongqing Academy of Science and Technology, Chongqing 401123, China

**Keywords:** engagement detection, body orientation estimation, multi-task learning, classroom analysis

## Abstract

Student engagement analysis is closely linked with learning outcomes, and its precise identification paves the way for targeted instruction and personalized learning. Current student engagement methods, reliant on either head pose estimation with facial landmarks or eye-trackers, are hardly generalized to authentic classroom teaching environments with high occlusion and non-intrusive requirements. Based on empirical observations that student body orientation and head pose exhibit a high degree of consistency in classroom settings, we propose a novel student engagement analysis algorithm incorporating human body orientation estimation. To better suit classroom settings, we develop a one-stage and end-to-end trainable framework for multi-person body orientation estimation, named JointBDOE. The proposed JointBDOE integrates human bounding box prediction and body orientation into a unified embedding space, enabling the simultaneous and precise estimation of human positions and orientations in multi-person scenarios. Extensive experimental results using the MEBOW dataset demonstrate the superior performance of JointBDOE over the state-of-the-art methods, with an MAE reduced to 10.63° and orientation accuracy exceeding 91% at 22.5°. With the more challenging reconstructed MEBOW dataset, JointBDOE maintains strong robustness with an MAE of 16.07° and an orientation accuracy of 88.3% at 30°. Further analysis of classroom teaching videos validates the reliability and practical value of body orientation as a robust metric for engagement assessment. This research showcases the potential of artificial intelligence in intelligent classroom analysis and provides an extensible solution for body orientation estimation technology in related fields, advancing the practical application of intelligent educational tools.

## 1. Introduction

Student classroom engagement analysis is a critical component of improving teaching quality, and it holds significant importance for achieving precision education [1,2]. Traditional manual observation methods suffer from limitations such as inefficiency and subjectivity, driving the trend toward automated detection technologies. Although current artificial intelligence and big data-based computer vision technologies have been widely applied in the educational field, the existing engagement analysis research primarily focuses on online classrooms, analyzing facial data and platform interaction data—and benefiting from the clear individual data collection in virtual environments [3,4]. However, in offline classroom teaching scenarios, methods relying on facial data analysis face significant challenges due to large class sizes and mutual obstructions among students. The mainstream engagement evaluation methods currently depend on identifying typical actions like raising hands or standing [5,6]. Yet, such approaches struggle to effectively assess engagement levels during the most common static listening states. On the other hand, humans’ body orientation usually reflects their attention directions, indicating a new solution for student engagement analysis in authentic classrooms [7].

Human body orientation estimation (HBOE), which involves estimating the orientation angles of humans, has been widely applied in fields such as human–computer interaction and intelligent monitoring [8,9]. However, its application within the educational domains remains relatively nascent. In this paper, we propose to leverage the HBOE method to analyze the student engagement during their listening status in classroom settings. It enables the tracking of individual learning states by detecting subtle changes in students’ body orientation, even in large-size classrooms with high occlusion. Additionally, this non-intrusive detection approach can meet the needs of large-scale routine classroom analysis, providing an innovative technical solution for implementing precision teaching.

However, most existing HBOE methods rely on a two-stage pipeline involving initial human detection, followed by orientation angle estimation. Such approaches are not only computationally intensive but also heavily dependent on the accuracy of the detection results. Previous HBOE studies have primarily focused on pre-cropped human instances under idealized conditions [10,11,12], limiting their generalization for practical applications. Therefore, developing an integrated and efficient HBOE solution is imperative.

In this paper, we propose a novel student engagement analysis method with one-stage HBOE, named JointBDOE, which integrates body detection and orientation estimation into one task. In particular, JointBDOE designs a unified embedding that integrates bounding box prediction and orientation angles, and it forms a single-stage end-to-end trainable HBOE framework. Moreover, the joint learning of body location and orientation angles enables shared human features to enhance orientation estimation, particularly in crowded and occluded classroom scenarios. With JointBDOE, all the students’ orientation directions are computed and displayed with arrow indicators for engagement analysis. Experimental results on reconstructed MEBOW and authentic teaching videos with various layouts demonstrate the effectiveness and efficiency of the proposed JointBDOE for multi-person orientation estimation and student engagement analysis, respectively. The main contributions are summarized as follows:

(1) Method innovation in intelligent classroom engagement analysis: Breaking through the limitation of traditional classroom engagement analysis that relies on manual observation or eye-tracker devices, we proposed a novel visual analysis method integrated with body estimation. This method maintains stable performance in crowded classroom scenarios with severe occlusions, and it fundamentally solves the core problem that traditional methods fail to adapt to the complex environment of real classrooms—realizing method-level innovation in adapting to practical classroom scenarios.

(2) Technological innovation of the One-Stage JointBDOE Framework: We develop a technologically innovative one-stage end-to-end JointBDOE framework to achieve the simultaneous collaboration of human body detection and orientation estimation. By integrating bounding box regression and orientation prediction into a unified feature embedding space and designing a decoupled orientation prediction head, this framework not only ensures real-time scalability but also significantly improves robustness against occlusion interference and viewpoint variations. It breaks through the technical bottleneck of existing multi-stage frameworks in which “step-by-step processing hinders the balance between efficiency and accuracy”, completing technological innovation in framework architecture.

(3) Application innovation in dataset and evaluation system: To solve the practical application problem that existing datasets lack diversity and cannot support real-scenario verification, we reconstruct and extend the MEBOW dataset to greatly enhance its scenario diversity and realism; meanwhile, we conduct comprehensive validation of JointBDOE across YOLOv5 and YOLOv11 models at multiple scales. Extensive experiments on benchmark data and authentic classroom videos confirm its superior accuracy and efficiency. Furthermore, it innovatively realizes a dynamic visualization of students’ attention directions via refined indicators, filling the application gap in intelligent classroom analysis for tools that integrate “quantitative analysis + intuitive presentation”—and providing practical application support for intelligent classroom management.

## 2. Literature Review

### 2.1. Student Engagement

Student engagement refers to the degree of attention, curiosity, interest, optimism, and enthusiasm that students demonstrate during learning, which also reflects their intrinsic motivation for academic progress [13,14]. Learning engagement is a critical element in the educational process, directly impacting students’ learning outcomes and developmental progress [15]. Research indicates that student engagement levels show positive correlations with academic performance, progress, graduation rates, satisfaction, and deeper learning. This relationship manifests not only in knowledge acquisition but also in students’ holistic development [16,17,18]. Especially in classroom teaching, a teacher faces a large number of students and finds it difficult to pay attention to each individual student. Therefore, an effective assessment of student participation cannot only concretely demonstrate the students’ classroom performance but must also provide a basis for the teacher to evaluate the effectiveness of classroom teaching.

The current research on student engagement primarily focuses on online learning environments. For instance, Liu et al. systematically investigated the impact of cognitive and affective engagement on learning outcomes in MOOC contexts [19]. Monkaresi et al. innovatively integrated facial expression analysis with self-report data to achieve engagement detection in online writing activities [20]. Rehman et al. established an online learning engagement assessment model through EEG signal analysis [21]. These studies employ diversified technological approaches that have significantly enhanced the objectivity, efficiency, and intelligence of engagement assessment. It is noteworthy that the successful implementation of these studies largely relies on the distinctive advantages of online learning environments, which enable precise acquisition of students’ facial feature data and comprehensive recording of learning behavior trajectories, thereby providing solid data support for engagement research.

However, classroom teaching remains the core front of education and a key link in improving education quality. Yet, due to issues like large class sizes, student occlusion, and the inability to use intrusive monitoring devices, it is difficult to clearly capture individual data like in online environments, making the direct transfer of online analytical techniques challenging. When facing dozens of students, teachers often struggle to monitor each student’s engagement status, making classroom participation assessment a prominent challenge in teaching practice. Common offline classroom engagement assessment methods include self-reporting and classroom observation. Self-reporting is simple and cost-effective but limited due to subjectivity and time delays, making it best for post-learning evaluations. Classroom observation using set scales records student performance, but both manual and semi-automated coding face high labor and time costs, limiting large-scale implementation. The emergence of computer vision technology provides new possibilities for automated classroom analysis. It can effectively capture teacher–student classroom behaviors, significantly reducing reliance on human resources while improving assessment efficiency. However, current technological applications mainly focus on identifying typical behaviors like hand-raising, yawning, phone use, and standing. In reality, during regular teaching processes, students spend most of their time in static listening states in which teachers find it hard to detect attention wandering. Moreover, in newly promoted teaching models like project-based learning, non-linear seating arrangements make behavior recognition particularly challenging.

### 2.2. Human Body Orientation Estimation (HBOE)

The human body orientation estimation (HBOE) is defined as estimating the skeletal orientation of a person at the orthogonal camera frontal view, which has been applied in various tasks, such as robotics navigation, intelligent surveillance, and human–computer interaction [22,23,24,25]. Early research primarily relied on handcrafted features and traditional classifiers, often modeling HBOE as a classification problem using simple multi-layer neural networks due to limitations in dataset scale and annotation accuracy. With the advancement of technology, the detection foundation has progressively evolved from traditional handcrafted feature-based approaches to deep learning-based methods, including Faster R-CNN [10], FCOS [11], and YOLOv5 [26]. Hara et al. laid the foundation for fine-grained orientation prediction by re-annotating the TUD dataset with continuous angle labels [27]. MEBOW further established a large-scale benchmark dataset and demonstrated the superiority of deep neural networks in HBOE tasks [25]. PedRecNet explored a multi-task learning framework that combines body orientation with 3D pose estimation, achieving competitive performance [28].

The current HBOE methods predominantly adopt a two-stage processing pipeline: first localizing human instances using pre-trained detectors (e.g., Faster R-CNN), followed by orientation estimation on the cropped regions. Wang et al. proposed Graph-PCNN, a two-stage human pose estimation method based on graph convolutional networks, which improves pose estimation accuracy through a graph-based pose refinement module [29]. Yang et al. proposed DWPose, which enhances the efficiency and accuracy of full-body pose estimation via a two-stage distillation strategy [30]. Lin et al. proposed a two-stage multi-person pose estimation and tracking method based on spatiotemporal sampling [31]. While straightforward and effective, this paradigm faces notable challenges in practical applications. On the one hand, instance cropping during detection may introduce information loss, particularly in cases of occlusion or dense crowds. On the other hand, the two-stage approach leads to computational costs that scale linearly with the number of individuals, making real-time performance difficult to achieve. In recent years, some studies have addressed the information loss and computational overhead of two-stage HBOE methods by proposing single-stage approaches. Wang et al. proposed YOLOv8-SP, an enhanced YOLOv8 architecture that integrates multi-dimensional feature fusion and attention mechanisms to achieve real-time pose estimation and joint angle extraction for moving humans [32]; Zhao et al. proposed Part-HOE, which estimates orientation using only visible joints and introduces a confidence-aware mechanism to improve robustness under partial observations [33]. However, although these single-stage methods improve computational efficiency and occlusion robustness, their performance in densely populated scenarios and real-world environments such as classrooms remains limited, and the precision, real-time capability, and generalization of the models still need further validation and optimization.

Moreover, most existing HBOE studies assume input to be precisely cropped human regions, significantly limiting their applicability in real-world scenarios. Multi-task learning strategies have gained considerable attention for their efficiency and potential for task synergy [34,35]. For instance, Raza et al. designed parallel CNN classifiers to separately predict head and body orientation [24]. MEBOW utilized body orientation as auxiliary supervision to enhance 3D pose estimation [25]. PedRecNet proposed a unified architecture for joint 3D pose and orientation estimation [28]. GAFA introduced a novel gaze estimation method leveraging the coordination between the human gaze, head, and body [36]. However, all of these studies rely on cropped human bounding box images as input, hindering their deployment in practical settings. Consequently, developing end-to-end methods capable of directly processing raw images while supporting multi-person orientation estimation remains a critical challenge for advancing this technology.

Compared with these advances, our proposed JointBDOE differs in two key aspects: (i) it integrates orientation estimation directly into the detection head, avoiding the person-dependent cost of two-stage approaches; and (ii) it is designed for real-time classroom scenarios, where robustness and scalability are as critical as accuracy. By situating our work within these latest developments, we show that JointBDOE complements recent trends while offering a practical solution tailored to multi-person classroom analysis.

## 3. Methodology for Student Engagement Analysis

We first propose a novel student engagement analysis method with body orientation estimation under the empirical observation that body directions usually indicate students’ attention. Then, to adapt to crowded and occluded authentic classroom settings, we integrate the body detection and orientation estimation into one task and design a one-stage end-to-end framework for multi-person body estimation. More details are introduced in the following subsection.

### 3.1. Overall Framework

**Figure 1:**
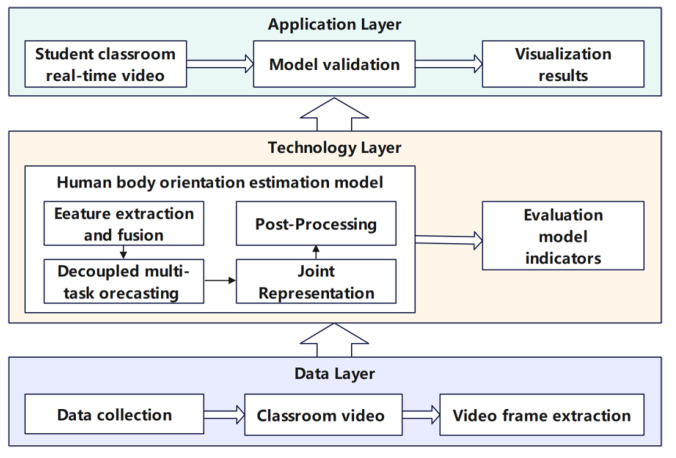
The proposed three-layer framework for intelligent student engagement analysis in authentic classrooms, including a data layer, a technology layer, and an application layer.

We propose a three-layer architecture for an intelligent student engagement analysis in authentic classroom settings, including data, technology, and application layers, as shown in Figure 1. First, the data layer collects learning state data with two cameras installed on the front and rear walls of the classroom. These continuous video streams are further converted into discrete frames with a sample rate of 3 s as the input for the following engagement analysis. Second, the technology layer integrates the proposed multi-person body orientation estimation method to identify all the students’ locations and body orientation angles in real time. Third, the application layer offers a real-time visual display, where bounding boxes and orientation narrows are represented for students and their orientation directions, respectively. To the end, the proposed architecture is successfully applied to analyze student engagement levels in authentic teaching with various layouts, including regular configuration, small-group discussions, and circular seminar configurations. Students’ engagement is intricately linked to their attention directions, which can be inferred from their body orientation. The frequency of their orientation changes and whether these changes are teacher-directed both indicate students’ engagement levels. The experimental section provides a detailed analysis of both patterns.

### 3.2. Joint Body Detection and Orientation Estimation (JointBDOE) Model

**Figure 2:**
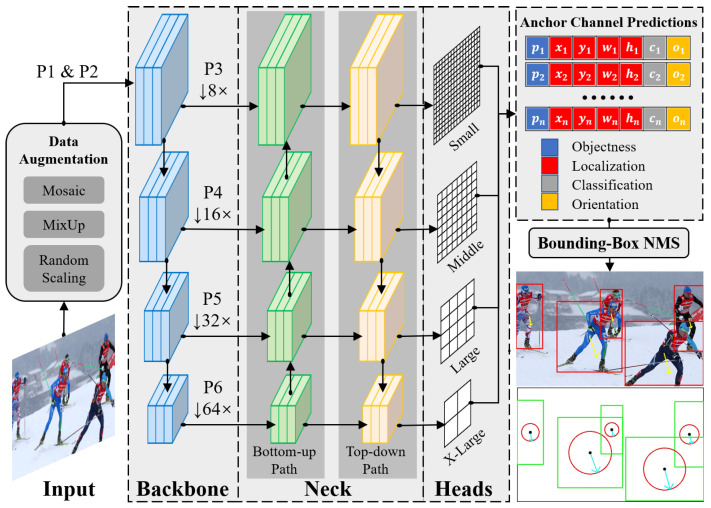
Our architecture builds upon YOLOv11 [37]. The input image is initially processed via the CSPDarknet53 [29] backbone to extract feature maps at four different scales P3, P4, P5, and P6. These multi-scale features are then fused using PANet [38] at the neck. The fused features are passed to detection heads, where each anchor channel produces a unified output that includes both bounding box and orientation predictions. The square frame represents the human body position, and the circular frame indicates the 360-degree orientation information of the human body on the 2D plane.

**Figure 3:**
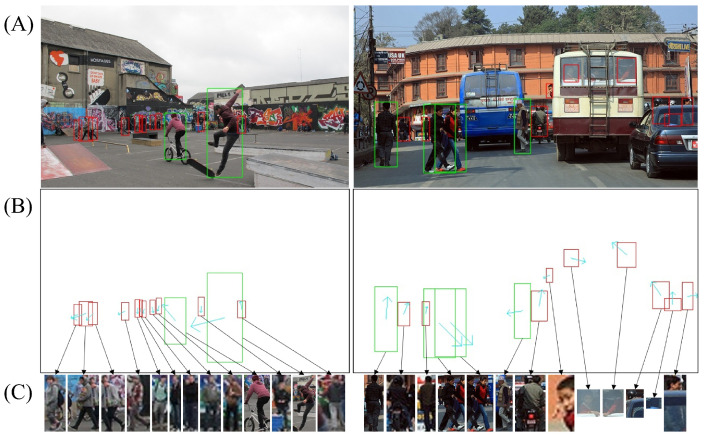
Two examples from the reconstructed MEBOW dataset [25]. (**A**): original crowd scenes, where green boxes are provided by MEBOW and red boxes are newly added by us. (**B**): body boxes with orientation arrows (cyan). (**C**): cropped human instances, including occluded, truncated, or small bodies. These samples are difficult for single-person HBOE methods but are effectively addressed by our single-stage multi-person approach.

To adapt to crowded and occluded classroom settings, we propose a novel single-stage end-to-end trainable framework that joint student detection and body orientation angles into one task, named JointBDOE, as shown in Figure 2. Combining the bounding box regression and orientation angle estimation into a unified embedding, JointBDOE integrates body orientation information into traditional object detection representation and transforms multi-person orientation estimation into a single-stage detection task. The overall architecture of JointBDOE is built upon the single-stage object detector YOLOv11 [37], which can be readily replaced with other single-stage architectures. In particular, with the input images/frames, we first employ the CSPDarknet 53 backbone [29] and PANet neck [38] for efficient feature extraction and fusion. Then, we design a decoupled prediction head with three independent branches, including classification, localization, and orientation estimation, to simultaneously detect human bodies and estimate orientation angles at various scales. Finally, non-maximum suppression (NMS) is applied to the prediction results and outputs with detected human positions and orientations. To facilitate joint learning, we reconstructed the MEBOW dataset [25], a representative dataset for body orientation estimation in the wild, with supplementing complete human body bounding box annotations and pseudo-orientation labels generated via Wu et al. [25]. Figure 3 shows several selected enhanced samples.

#### 3.2.1. Unified Embedding

To build a single-stage human detection and orientation estimation framework, we propose a unified embedding representation method to integrate the two tasks. This approach extends traditional object detection representations by incorporating human-related attributes into a unified expression [39]. Specifically, the unified embedding is defined as e=(p,x,y,w,h,c,o), where *p* denotes the probability of target existence (i.e., human presence), and (x,y) represents the center coordinates of the human bounding box, with *w* and *h* indicating its width and height—these four parameters collectively determine the precise spatial location of humans in images. *c* stands for classification scores reflecting the confidence level of detection results belonging to the human category, and *o* indicates the orientation of the human. Through this representation, the originally complex multi-person orientation estimation problem is successfully transformed into a conventional object detection task. The primary advantage of this design lies in enabling the model to simultaneously learn multiple related tasks with a minimal computational cost by sharing a single network head. This approach not only significantly improves the model’s operational efficiency but also remarkably enhances task processing capability, allowing the entire system to maintain high performance while achieving more efficient deployment and application.

In the implementation, we extend the anchor prediction mechanism of YOLOv11 by working with the output group $\hat{H_{i}^{s}}$ for the *i*-th image grid cell at scale reduction factors s∈{8,16,32,64}. In YOLOv11, each detection head has a fixed number of anchor channels, $N_{a}=$ 3. For a specific anchor channel prediction, ${\hat{H}}_{i,a}^{s}$, its original representation is $\left(\hat{p},\hat{t},\hat{c}\right)$, which includes the objectness score $\hat{p}$, bounding box offsets $\hat{t}=\left(\hat{x},\hat{y},\hat{w},\hat{h}\right)$, and classification scores $\hat{c}=\left({\hat{c}}_{1},\cdots ,{\hat{c}}_{k}\right)$. For the human body orientation estimation task, we maintain the single-class classification setting (k=1) and augment the original output with an orientation parameter $\hat{o}$, thereby constructing the complete prediction embedding $\hat{e}=\left(\hat{p},\hat{x},\hat{y},\hat{w},\hat{h},\hat{c},\hat{o}\right)$. This unified embedding design not only preserves the functionality of the original detection framework but also allows flexible extension to other tasks requiring simultaneous prediction of object attributes and locations. For instance, by replacing $\hat{o}$ with Euler angle parameters, the framework can be readily applied to tasks such as eye gaze direction and head pose estimation.

#### 3.2.2. Decoupled Head

**Figure 4:**
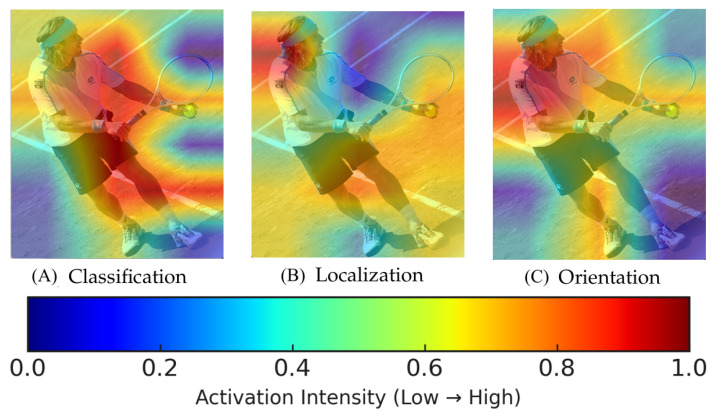
Areas focused on by different tasks. The classification task focuses on the whole body area, the localization task focuses on coordinate positions of the bounding box, and the orientation task focuses on upper half of the body. Warmer colors (red/yellow) indicate higher attention/activation of the corresponding branch, and cooler colors (blue) indicate lower attention.

**Figure 5:**
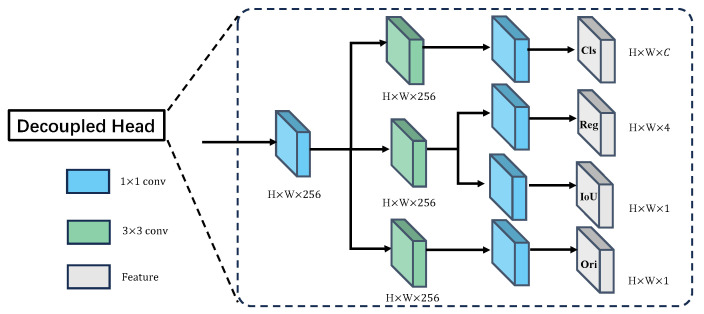
Feature map obtained from PANet [38] is fed into the decoupling head. First, it goes through a 1×1 convolution block for feature dimension reduction, resulting in a H×W×256 feature map. It then passes through three parallel 3×3 convolution layers, one for classification, one for localization and confidence estimation, and one for orientation estimation. Finally, each of them goes through a 1×1 convolution for four tasks, respectively.

During multi-task joint training, we observed that the features extracted for the three distinct tasks of classification, localization, and orientation estimation emphasize different aspects. The three heatmaps shown in Figure 4 demonstrate that the features for classification cover the entire human body, and the features for localization focus on regions that refine bounding box coordinates, while the features for orientation estimation primarily concentrate on the upper body portion. The discrepancies arising from learning different tasks from the same features may lead to slower convergence and performance degradation. To address this issue, we decoupled the head module into three independent branches, each dedicated to a specific task, as illustrated in Figure 5.

#### 3.2.3. Body Orientation Estimation

In this paper, human body orientation is defined as a continuous angle, θ∈[0,360), representing the orientation of the human body relative to the camera’s frontal view. In the proposed unified embedding vector, e=(p,x,y,w,h,c,o), the orientation component *o* is normalized to the [0,1) range via the sigmoid activation function. This processing not only facilitates network training optimization but also effectively enhances the model’s robustness to angular features. During the training phase, we adopt the standard mean squared error (MSE) for orientation regression. To address the boundary discontinuity issue caused by angle periodicity, a wrapped MSE loss function is introduced. When calculating errors, it accounts for the periodic nature of the angular space to optimize the supervision mechanism. Since the periodicity of azimuth angles can mislead the original difference between predicted and true values near the 0/2π boundary, the second term in Equation (1) ensures the accurate measurement of the minimum angular difference by adapting to this periodicity. Its specific form is as follows:

(1) $$L_{ori}=\frac{1}{n}\sum_{i=1}^{n}\min(∥{\hat{o}}_{i}-o_{i}{∥}_{2},∥1-|{\hat{o}}_{i}-o_{i}{|∥}_{2}),$$

where ${\hat{o}}_{i}$ is the estimated result from *i*-th multi-scale head, $o_{i}$ is the corresponding ground-truth. Here, n denotes the number of multi-scale prediction heads; we set n=4 following prior work (e.g., MEBOW).

**Figure 6:**
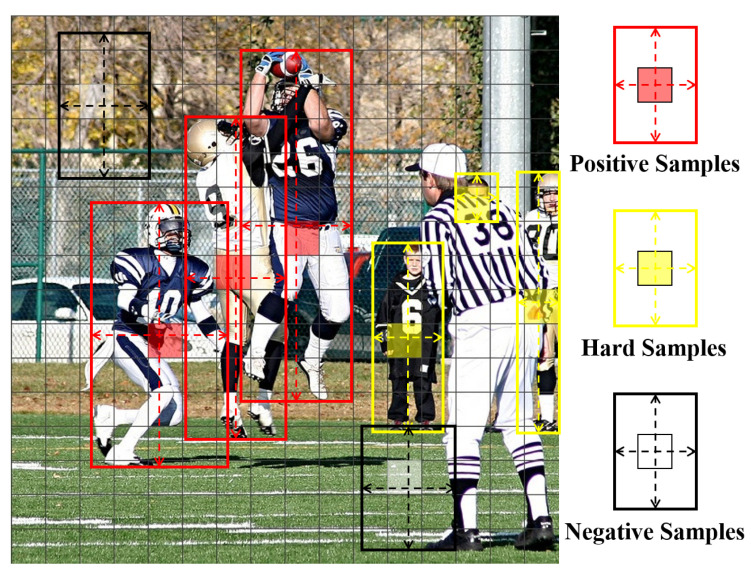
The illustration of some anchor channel predictions for positive, hard, and negative body orientation regression. This was provided as an example for illustration only.

Although human-body orientation appears intuitive and straightforward in images, we have identified two potential challenges for this task. First, for severely occluded, highly truncated, or extremely small human instances, their orientation is often difficult to determine accurately. Second, dense anchor channel predictions may contain numerous areas with partial human body or none. Figure 6 presents an illustration. These special samples have a limited or no effect on the supervised learning of body orientation. Thus, we designed a probability threshold τ-based filtering mechanism to screen $\hat{p}$, effectively eliminating unreliable orientation estimates ${\hat{o}}_{i}$ from the prediction results. However, false-negative hard samples should be retained, as they play a crucial role in the joint estimation task, as shown in the bottom display of Figure 3. A suitable value for τ is obtained via ablation studies.

(2) $$L_{ori}^{\prime }=\sum_{s}\phi \left(\hat{p}>\tau \right)\min(∥\hat{o}{-o∥}_{2},∥1-|\hat{o}-{o|∥}_{2}),$$

where ϕ(·) is an indicator function that equals 1 for person instances with valid orientation labels and 0 otherwise, so that the orientation loss is applied only when annotations are available.

#### 3.2.4. Overall Loss Function

Under the unified embedding, the multi-person body estimation can be solved through the following function:

(3) $$L=\alpha L_{obj}+\beta L_{box}+\lambda L_{ori}^{\prime },$$

where $L_{obj}$ and $L_{box}$ are the original detection losses for objectness and localization, and $L_{ori}^{\prime }$ is the orientation estimation. The settings of weight parameters α=0.7 and β=0.05 are the same in YOLOv11. The optimal value of λ for orientation regression loss is explored through ablation studies.

The definitions of $L_{obj}$ and $L_{box}$ are as follows:

(4) $$L_{obj}=\frac{1}{n}\sum_{i=1}^{n}\mathrm{BCE}\left(\hat{p},p\cdot \mathrm{CIoU}\left({\hat{t}}_{i},t_{i}\right)\right),$$

and

(5) $$L_{box}=\frac{1}{n}\sum_{i=1}^{n}\left[1-\mathrm{CIoU}({\hat{t}}_{i},t_{i})\right],$$

where BCE is the binary cross-entropy, and CIoU is the complete intersection over union. The body objectness p=1 is multiplied by CIoU score to promote concentrated anchor channel predictions. And p=0 means no target human body.

## 4. Experiments

We first demonstrate the effectiveness and efficiency of the proposed JointBDOE model on the multi-person body orientation task on both the original MEBOW dataset and its reconstructed version, where the reconstructed one contains more challenging and complex crowded samples. Then, we test the performances of JointBDOE for student engagement analysis in authentic classroom settings with various layouts, and we dynamically visualize students’ engagement levels during classes.

### 4.1. Datasets and Metrics

Reconstructed MEBOW Dataset: The MEBOW dataset contains a total of 54,007 annotated images, including 51,836 images (127,844 human instances) for training and 2171 images (5536 human instances) for testing. While preserving the original data, we restored the challenging human instances originally provided through the COCO [40] dataset and employed the MEBOW method to generate pseudo labels for body orientation. The final constructed dataset comprises 225,912 human instances (216,853 for training and 9059 for testing), suitable for multi-person body orientation estimation tasks.

Metrics: Following [25,28], this paper employs two core metrics to evaluate body orientation estimation performance: first, the mean absolute error (MAE) measuring the average deviation between predicted angles and ground truth; second, the Acc.-X° accuracy metric (X∈{5,15,30}), which indicates the proportion of predictions falling within an *X*° tolerance range centered on the ground truth orientation. For the joint body detection task evaluation, the recall metric is additionally reported as a supplementary reference. Through these metrics, we can comprehensively evaluate the model’s overall performance in both body orientation estimation and human body detection tasks, thereby validating the robustness and practical value of the proposed method in complex scenarios.

### 4.2. Implementation Details

This study employs the YOLOv11 architecture as the backbone network, replacing the standard detection head with our designed three-branch decoupled detection head (shown in Figure 5), while maintaining the basic training configurations from reference [41]. Utilizing the proposed unified feature embedding approach, the system simultaneously accomplishes both human body detection and orientation estimation tasks. All input images are standardized to a 1024×1024 resolution (preserving original aspect ratios), with training parameters τ and λ manually fine-tuned through experimental validation. All experiments are run on a workstation with six NVIDIA GeForce RTX 4090 GPUs (24 GB each), driver 550.78, and CUDA 12.4. Unless otherwise noted, we train on four GPUs using torchrun with a global batch size of 32 (8 images/GPU). Our implementation uses Python 3.10.4 and PyTorch 2.6.0 within the YOLO framework. We adopt SGD with an initial learning rate of $1.0\times 10^{-2}$, momentum of 0.937, and weight decay of $5.0\times 10^{-4}$. A OneCycleLR scheduler is used with a final LR ratio 0.2, preceded by a 3-epoch warm-up starting at 0.1× the base LR. Training lasts 500 epochs with mixed precision (AMP) and EMA of weights (decay 0.9999). Input images are resized to 1024×1024 and augmented with standard YOLO policies. Mosaic is enabled with a probability of 1.0 throughout training, mixup is disabled, and flips (horizontal and vertical) are disabled. Other augmentations, including HSV jitter (h = 0.015, s = 0.7, v = 0.4), random translation (±0.1), scaling (0.9×), rotation, shear, perspective, and copy–paste, are disabled. Random seeds are initialized for each training process to ensure reproducibility. The best-validation checkpoint is selected with mAP@0.5:0.95. When the global batch size changes, the base learning rate is scaled linearly.

### 4.3. Results on Multi-Person Body Orientation Task

#### 4.3.1. Ablation Studies

In previous studies [42], we trained the yolov5s model for 500 epochs. As shown in Table 1, we initially set λ to 0.1 and screened the threshold τ within the range of [0.1, 0.4] at intervals of 0.1. The experimental results indicate that the lowest MAE value was achieved when τ was set to 0.2, demonstrating the importance of reasonable hard-sample filtering. Subsequently, with τ fixed, we selected the loss weight λ for $L_{ori}^{\prime }$ from {0.02,0.05,0.10,0.15} and ultimately determined that λ= 0.05 achieved the optimal balance between human detection and orientation estimation tasks. Building on this, we upgraded the backbone network from YOLOv5 to YOLOv11 and conducted training in the same manner. The results (Table 1) show that under identical conditions (τ= 0.2, λ= 0.05), the YOLOv11 backbone network exhibits superior performance. This also indicates that our method is not limited to a specific backbone network but, rather, exhibits a strong generalization capability and adaptability.

Meanwhile, we conducted dedicated ablation experiments on the decoupled head structure to verify its effectiveness. In our previous study [42], based on the YOLOv5 architecture, we demonstrated through a comparative analysis of the performance differences between coupled and decoupled head structures that the decoupled design can effectively reduce feature interference between multiple tasks, thereby achieving superior performance (Table 2). Specifically, the Decoupled-YOLOv5 model showed improved direction estimation accuracy and higher accuracy within the 5° error range across all backbone network versions while maintaining an unchanged detection recall rate. Among them, YOLOv5l performed the best, achieving a mean absolute error of 10.946° in direction estimation and an accuracy rate of 47.8% within 5° error range. Considering the continuous updates of backbone networks, we further tested the latest YOLOv11 architecture. The results showed that the Decoupled-YOLOv11x model achieved even better performance, reducing the mean absolute error of direction estimation to 10.631° and increasing the accuracy within 5° error range to 48.7%. The results further confirm the superior performance of our proposed decoupled head structure in orientation estimation tasks, while demonstrating its strong generalization capability across different backbone network architectures.

#### 4.3.2. Quantitative Comparison

For a fair comparison, we first evaluated model performance on the original MEBOW dataset, which excluded numerous challenging instances. As shown in Table 2, the YOLOv5l-based model achieved comparable results to the MEBOW and PedRecNet baseline methods in terms of mean absolute error (MAE) and accuracy metrics, while the subsequently adopted YOLOv11 model further improved these metrics. These baseline methods were specifically designed for single-person body orientation estimation tasks and required precisely cropped human instances as input. Notably, in multi-target scenarios without predefined human detection, the MEBOW and PedRecNet methods demonstrated limited effectiveness, whereas our approach maintained superior performance.

Furthermore, we conducted a validation of the proposed method’s performance on the reconstructed MEBOW dataset, with detailed experimental results presented in Table 3. Compared to the original dataset, the reconstructed version incorporates more challenging real-world samples (e.g., severe occlusion, low-resolution cases). While these challenging samples led to some degree of performance degradation in the overall metrics, these results objectively reflect the technical difficulties encountered in practical application scenarios. These carefully constructed, challenging samples also provide a more realistic test benchmark for future research, which will effectively advance the development of in-the-wild human body orientation estimation technology.

In addition, to comprehensively present the computational cost of the model, we also report the number of parameters (Params) and floating-point operations per second (FLOPs). For the two-stage baseline models (MEBOW and PedRecNet), we report a composite computational cost, which is equal to the FLOPs of the detector per image plus the product of the FLOPs for orientation estimation per person and the average number of persons in an image. For the single-stage YOLO series models, we directly report the number of parameters and FLOPs corresponding to each 1024 × 1024 image. The relevant results are shown in Table 4.

#### 4.3.3. Qualitative Comparison

**Figure 7:**
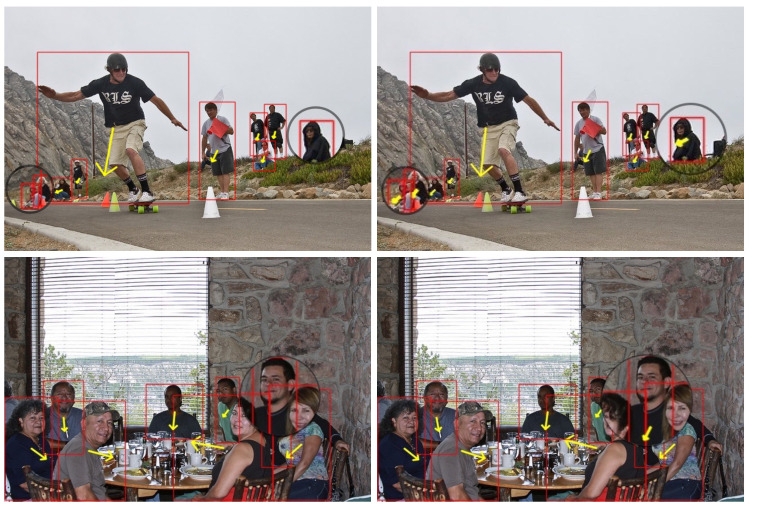
The results on extended MEBOW data. The first column is estimated through MEBOW [25], and the second column is estimated using our method. The red boxes indicate human targets, and the yellow arrows represent body orientation. The yellow arrow is drawn from the bounding box center, where the direction corresponds to the orientational angle. The length of the yellow arrow is fixed relative to the box size for visualization clarity.

Figure 7 presents the qualitative evaluation results of our method in various complex scenarios. The predicted orientation o∈[0,1] is linearly mapped to an angle, θ∈[0,2π) by θ=2π·o, for visualization. The yellow arrow is drawn from the bounding box center in the direction of θ, and its length is fixed relative to the box size for clarity. Comparative results demonstrate that our method effectively addresses the detection failures (indicated with black circles) observed in MEBOW under crowded and occluded scenarios. It can be observed that our approach demonstrates remarkable robustness in real-world situations with dense crowds and severe occlusions. Not only can it effectively detect human bodies in low-quality image regions, but it also accurately predicts their orientation angles, fully showcasing the algorithm’s strong adaptability in practical application scenarios.

**Figure 8:**
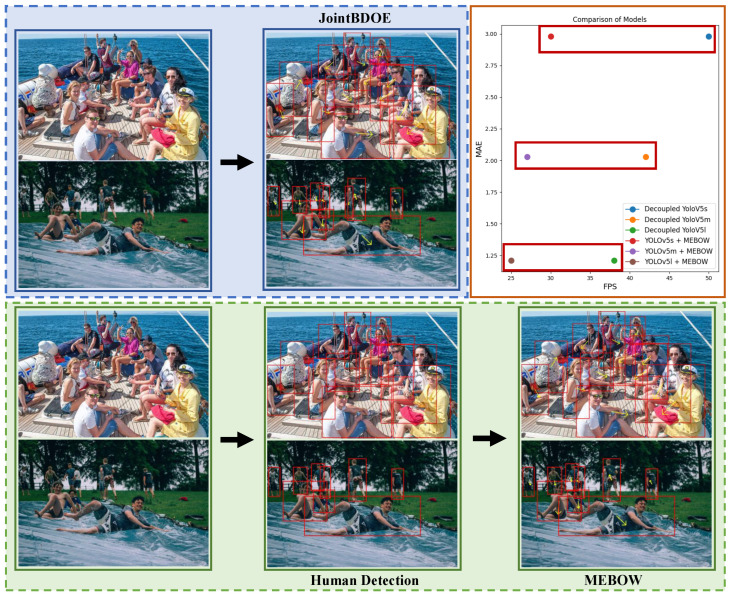
The first row delineates the workflow of our method, while the second row represents MEBOW [25]. The scatter plot in the upper right corner visually compares the FPS and MAE between JointBDOE and MEBOW. The term MAE specifically denotes the difference in the estimation results of JointBDOE and MEBOW when employing an identical detection model. The square frame represents body position, and the arrow indicates its facing direction.

Figure 8 presents a comprehensive comparative analysis between the proposed JointBDOE framework and the conventional MEBOW approach. JointBDOE adopts an innovative end-to-end design concept, requiring only single-stage processing to directly output precise orientation estimation results from raw input images, significantly simplifying the traditional workflow. In contrast, the MEBOW method requires first obtaining human bounding boxes through external detection algorithms (such as Faster RCNN or YOLO) as input before performing subsequent orientation estimation. This two-stage architecture not only increases computational overhead but also introduces additional error accumulation risks. The scatter plot in the upper right corner further confirms that, under the same baseline detection framework, JointBDOE achieves comparable estimation accuracy to MEBOW while operating at a faster processing speed. This makes the algorithm particularly valuable for applications with high real-time requirements.

### 4.4. Results on Student Engagement Analysis

In the educational domain, public datasets with students are extremely limited due to ethical and privacy concerns. To address this issue, we construct an authentic classroom teaching dataset from cooperated schools for student engagement analysis. The dataset comprises 10 complete classroom recording videos, each approximately 40 min in duration. The distinctive characteristics of the constructed dataset include the following: (1) various layouts and spatial configurations; (2) diverse camera positions and perspectives; (3) a wide age range from kindergarten to university; and (4) differential seating arrangements, including row–column patterns and group discussions. These characteristics enable the constructed dataset to comprehensively characterize classroom teaching scenarios, thus serving as an effective validation resource for student engagement analysis in classroom settings. In compliance with privacy protection principles, we implement rigorous blurring processing on all facial information of teachers and students in the videos.

**Figure 9:**
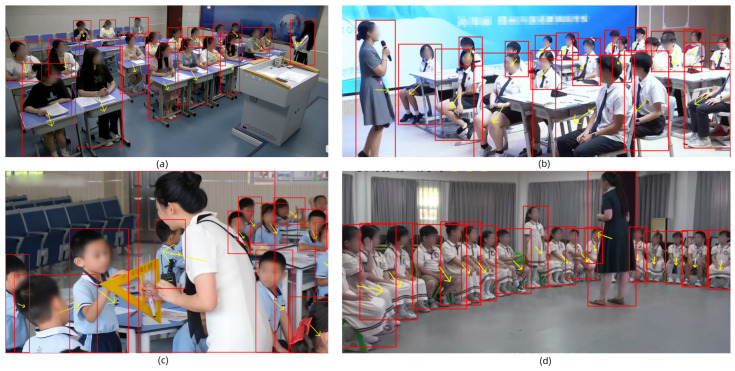
Visualized results of JointBDOE on student detection and their body orientation estimation in various classroom layouts. (**a**) Row–column seating arrangement, (**b**) group discussion seating arrangement, (**c**) teacher–student interaction, and (**d**) circular seating arrangement. The square frame represents body position, and the arrow indicates its facing direction.

Figure 9 shows the results of student/teacher detection and orientation estimation under various seating arrangements and camera perspectives, where red bounding boxes represent detected teachers and students, and yellow arrows indicate their body orientations. The visualized results demonstrate that the proposed JointBDOE can not only detect almost all the students but also accurately estimate their orientations, even in complex scenarios with crowded students and severe occlusions. From Figure 9, we observe that students’ attention directions are almost consistent with their body orientations; thus, student engagement can be inferred from their body orientations. Meanwhile, JointBDOE can simultaneously identify teachers and their orientations, which supports for mining effective teacher–student interactive patterns.

**Figure 10:**
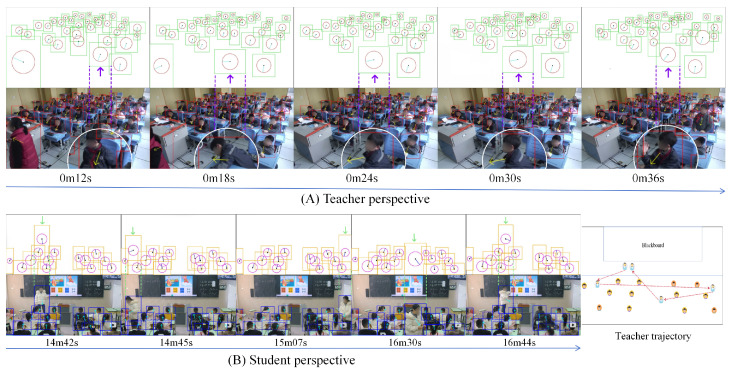
Example of continuous course visualization. The square frame represents body position, and the arrow indicates its facing direction.

Furthermore, we demonstrate how to use students’ body orientation for engagement analysis based on continuous teaching segments. Figure 10 provides dual-perspective visualizations of body orientations from both the teacher and students, along with simplified diagrams above to enhance readability in crowded scenarios. In case (A), with the teacher perspective of Figure 10, when the teacher turns to write on the blackboard, most students maintain high engagement as their body orientations focus on the teacher/blackboard. Meanwhile, the student pointed by the purple arrow constantly changes his orientation, indicating slight disengagement. Combined with timestamp information (class just begins), this phenomenon may indicate that the student has relatively weak self-discipline and has not yet fully entered a focused learning state during the initial class phase. In case (B), with the student’s perspective of Figure 10, based on the size and position of the bounding box, it can be inferred that the person pointed to with the green arrow is the teacher. Between 14 and 16 min of class, the teacher moved back and forth from the podium to the students’ area. Such a movement pattern indicates that the teacher assigns a task and then walks to the students, checking their progress. During this period, all the students hardly changed their orientation, demonstrating a high level of engagement, while the teacher’s movement path showed attention to each row of students.

To sum up, the proposed JointBDOE achieves excellent performances on orientation estimations of teachers and students in various scenarios, including front-view, back-view, and crowded settings. Moreover, the dynamic changes in orientation reveal students’ engagement levels and can also provide objective evidence for dividing classroom teaching segments. This offers strong support for instructional analysis and personalized guidance, fully demonstrating its practical value in educational settings.

### 4.5. Limitations and Future Research

Although our proposed learning engagement recognition method has achieved good results in various classroom scenarios, there is still room for further improvement. On the one hand, students’ orientation and position are important cues for evaluating classroom engagement, but typical behaviors (such as raising hands or standing up) are also of critical importance in actual teaching, especially in dynamic or collaborative learning situations where interactions and movements are more complex. On the other hand, the dataset used in this study is mainly based on visual information, whereas teacher–student verbal interaction in real classrooms also has a significant impact on teaching effectiveness. In addition, the current engagement detection results are mainly presented in a technical visualization form and have not been fully integrated with educational theory, which limits their interpretability and instructiveness.

Future research will advance in several directions. First, at the feature level, we will integrate the recognition of regular and typical behaviors, including scenarios with more dynamic and collaborative interactions, and combined with students’ position coordinate encoding, to achieve a comprehensive engagement analysis at both the individual and group levels. Second, at the data level, we will incorporate multimodal information such as audio and video to build a more comprehensive classroom evaluation system. Third, at the model level, we will further enhance the performance of JointBDOE in complex scenarios based on its single-stage, low-computational-cost advantages, and explore adaptive optimization strategies for different educational environments. Fourth, at the application level, we will deeply integrate detection results with educational theory to provide teachers with more interpretable and instructive feedback, thereby promoting the innovation and practical implementation of education quality evaluation systems. Through these improvements, we aim to further enhance the accuracy, applicability, and practical value of engagement assessment while maintaining the advantages of being non-intrusive and automated.

## 5. Conclusions

This study has innovatively proposed a classroom engagement detection method based on human body orientation estimation and developed a model named JointBDOE to efficiently achieve human body orientation estimation. Through an end-to-end architecture, we successfully integrated object detection with orientation estimation, effectively addressing the adaptability and computational efficiency challenges in multi-person scenarios. Given the current scarcity of publicly available classroom datasets, we conducted experimental validation on the MEBOW dataset while performing further testing through reconstructed MEBOW datasets and self-collected real classroom teaching video datasets. The results confirm the effectiveness and broad applicability of our method. This research provides new approaches and robust technical support for classroom analysis and teaching quality assessment in the education field. With technological advancements and evolving educational needs, we will expand the dataset scale and optimize the experimental design to further validate the method’s applicability across different educational scenarios. 

## Figures and Tables

**Table 1:** Influence exploration of two hyperparameters: the tolerance threshold τ and the loss weight λ for $L_{ori}^{\prime }$. YOLO-s refers to the small variant of the YOLO detector. Upward arrows denote that higher values are preferable and downward arrows denote that lower values are preferable.

Backbone	τ	λ	MAE↓	Acc.-5°↑	Acc.-15°↑	Acc.-30°↑	AP^0.5^↑	AP^0.5:0.95^↑
yolov5s	0.1	0.1	13.848	43.60	79.29	90.83	82.9	59.0
yolov5s	0.2	0.1	13.758	43.02	79.02	90.67	82.8	58.7
yolov5s	0.3	0.1	14.493	41.98	77.91	89.68	82.3	58.1
yolov5s	0.4	0.1	15.122	40.66	76.84	89.69	81.7	57.4
yolov11s	0.1	0.1	13.852	42.05	78.47	90.77	83.3	58.8
yolov11s	0.2	0.1	13.611	42.25	78.50	90.76	83.2	58.6
yolov11s	0.3	0.1	14.089	41.87	78.33	90.60	83.2	58.2
yolov11s	0.4	0.1	14.399	41.44	77.14	89.80	82.8	57.9
yolov5s	0.2	0.02	13.738	41.44	79.18	90.88	84.8	62.0
yolov5s	0.2	0.05	13.427	42.88	79.64	91.21	84.1	61.1
yolov5s	0.2	0.10	13.758	43.02	79.02	90.67	82.8	58.7
yolov5s	0.2	0.15	14.715	41.08	77.21	89.96	80.5	55.7
yolov11s	0.2	0.02	13.743	41.92	78.20	90.42	84.6	61.1
yolov11s	0.2	0.05	13.346	41.72	78.76	91.11	84.2	60.5
yolov11s	0.2	0.10	13.949	41.02	78.39	90.34	82.8	58.1
yolov11s	0.2	0.15	14.733	40.05	76.48	89.65	81.0	55.6

**Table 2:** Body orientation performance comparison of our method with SOTA methods in the MEBOW dataset. The “–” means not reported. C- denotes coupled method and Dec- is decoupled method. YOLO-s/m/l/x refer to the small, medium, large, and extra-large variants of the YOLO detector, respectively. Upward arrows denote that higher values are preferable and downward arrows denote that lower values are preferable.

	Methods	MAE↓			Accuracy↑			Recall↑
			5°	15°	22.5°	30°	45°	
Single-person	MEBOW [25]	8.393	68.6	90.7	93.9	96.9	98.2	–
	PedRecNet [28]	9.700	–	–	92.3	–	97.0	–
Multi-person	C-YOLOv5s	13.130	44.1	89.9	88.1	91.8	94.2	99.55
	C-YOLOv5m	11.907	46.4	83.0	89.7	92.9	95.4	99.49
	C-YOLOv5l	11.207	47.5	83.4	90.5	93.8	96.0	99.46
	Dec-YOLOv5s	12.714	44.9	80.4	88.5	92.0	94.9	99.65
	Dec-YOLOv5m	11.073	46.9	83.4	90.0	93.8	96.3	99.52
	Dec-YOLOv5l	10.946	47.8	83.8	90.9	94.0	96.5	99.50
	Dec-YOLOv11s	13.43	40.5	79.1	87.8	91.5	94.8	99.64
	Dec-YOLOv11m	12.6496	43.7	80.2	88.3	91.8	94.9	99.57
	Dec-YOLOv11l	11.317	45.5	82.6	90.0	93.3	96.0	99.53
	Dec-YOLOv11x	10.631	48.7	84.4	91.2	94.0	96.4	99.57

**Table 3:** Our performance on the reconstructed MEBOW dataset with more crowded and complex samples. Upward arrows denote that higher values are preferable and downward arrows denote that lower values are preferable.

Methods	MAE↓	Accuracy ↑			AP↑	Recall↑
		5°	15°	30°		
Coupled-YOLOv5s	20.670	37.0	69.7	83.5	84.4	97.37
Coupled-YOLOv5m	18.763	39.8	73.5	85.5	85.8	97.63
Coupled-YOLOv5l	17.848	40.8	74.4	86.5	86.1	97.57
Decoupled-YOLOv5s	18.988	37.98	71.0	84.7	84.6	97.68
Decoupled-YOLOv5m	17.921	40.3	73.8	86.2	85.9	97.83
Decoupled-YOLOv5l	17.032	41.5	74.9	87.1	86.4	97.94
Decoupled-YOLOv11s	20.939	34.4	69.2	83.5	84.1	97.37
Decoupled-YOLOv11m	20.087	36.9	70.4	84.1	85.4	97.68
Decoupled-YOLOv11l	17.544	39.7	74.2	87.0	85.8	97.56
Decoupled-YOLOv11x	16.074	43.4	77.2	88.3	85.1	97.76

**Table 4:** Computational efficiency comparisons under the metrics of the number of parameters (Params) and FLOPs.

	Model		Params (M)	FLOPs (G)
Two-stage	MEBOW		72.1	446.5
	PedRecNet		74.5	456.4
Single-stage	JointBDOE	YOLOv5s	9.5	76.5
		YOLOv5m	24.5	210.6
		YOLOv5l	49.8	461.7
		YOLOv11s	11.9	100.3
		YOLOv11m	22.7	286.8
		YOLOv11l	28.3	371.1
		YOLOv11x	58.1	805.5

## Data Availability

Data can be obtained from the first author upon request.

## Abbreviations

The following abbreviations are used in this manuscript:

HBOE	Human Body Orientation Estimation
MOOC	Massive Open Online Course
EEG	Electroencephalography
MAE	Mean Absolute Error
CNN	Convolutional Neural Network
NMS	Non-Maximum Suppression
